# Estimates of average energy requirements in Bangladesh: Adult Male Equivalent values for use in analyzing household consumption and expenditure surveys

**DOI:** 10.1016/j.dib.2017.07.022

**Published:** 2017-07-17

**Authors:** Jillian L. Waid, Jessica R. Bogard, Shakuntala H. Thilsted, Sabine Gabrysch

**Affiliations:** a Institute of Public Health, Heidelberg University, Heidelberg, Germany; b Helen Keller International, Dhaka, Bangladesh; c School of Public Health, The University of Queensland, Brisbane, Australia; d Agriculture Flagship, Commonwealth Scientific and Industrial Research Organisation (CSIRO), Brisbane, Australia; e WorldFish, Greater Mekong Region, No. 35, Street 71, Sangkat Boeung Keng Kang 1, Khan Chamkar Morn, Phnom Penh, Cambodia

**Keywords:** Bangladesh, Food consumption, Household consumption and expenditures surveys, Energy requirements, Adult Male Equivalent (AME)

## Abstract

This dataset contains Adult Male Equivalent (AME) values for use in Bangladesh. These were constructed using prescriptive nutritional constructs adapted to the actual growth and weight pattern seen in Bangladesh. This dataset provides a common base to facilitate for future work with household consumption and expenditure data in Bangladesh while updating the average energy requirements for infants and young children for the WHO 2006 growth standards and 2007 growth reference curves.

**Specifications Table**

**Table:** 

Subject area	*Public Health*
More specific subject area	*Nutrition; economics*
Type of data	*Table of average energy requirements and corresponding adult male equivalents*
How data was acquired	*Updating energy intake methods through analysis of existing datasets*
Data format	*Tables in an Excel workbook*
Experimental factors	*Not applicable*
Experimental features	*Not applicable*
Data source location	*Global (for children 0–9 years of age) and Bangladesh (adolescent and adults 10–100 years of age)*
Data accessibility	*The data are stored with this article*

**Value of the data**

• The dataset provides a resource for future work with household consumption and expenditure surveys in Bangladesh.
• The prescriptive nutritional requirements used in the construction of this dataset reflect the energy requirements of a healthy population.
• The methods used to scale the adolescent and adult energy requirements could be applied to other countries.
• A standard definition of AME within Bangladesh will enable better comparison of results across researchers working with the same or similar datasets.

## 1 Data

The Excel workbook provides the weight, estimated energy requirement (in both kcal and kJ), and adult male equivalents (AME) for the both sexes and all ages in the Bangladeshi population. For children and adolescents, 0–18 years of age, the data are presented by month and year of age separately for each sex. For adults, the data are presented in three age ranges (19–29 years, 30–59 years, and 60+ years) separately for each sex. In addition, supplemental adjustments for pregnancy and lactation are provided.

## 2 Experimental design, materials and methods

Existing AME in the literature use the out-of-date NCHS 1977 growth reference for children and universal standards of adult height that may not match adult growth in a country [1], [2], [3]. We calculated the AME unit to include the child growth pattern recorded in the 2006 growth standards and 2007 growth reference curves for children, and based the adult energy requirements on the current average physical stature of Bangladeshis using FAO guidelines [4], [5], [6], [7]. We calculated AMEs using prescriptive nutritional constructs, providing smoothed energy requirements between child growth standards and observed measurements in adults. Adjustments for observed adult stature are important as excess energy intake at these ages will lead to adiposity and are not needed for adequate nutrition. Specific details of the methods used by age are available in Table 1 (organized by age).

**Table 1:** Details of energy requirements calculations by age group.

Age in completed years	Calculation instructions (Food and Agriculture Organization of the United Nations, 2001)	Calculation of energy requirement
0	The manual requires two parameters to estimate energy requirements, weight and growth, and the formulas vary by sex and age. Table 3.1 (page 13) provides eight formulas to measure the energy requirements of growth depending on the child's age (0–2 m, 3–5 m, 6–8 m, and 9–11 m) and sex. A single formula is used for total energy expenditure and is given at the bottom of table 3.2 on page 14 (no differentiation by sex).	The mean daily weights of infants 0 to 365 days were calculated using the 2006 WHO growth standards for weight for age. Optimal growth per day was calculated by subtracting the mean weight of an infant from the mean weight of an infant of the same sex who is one day older. Using FAO formulas, daily energy requirements for infants of each day of age and sex were calculated. Daily energy requirements were averaged to obtain the average energy need for each month of life and averaged over the year as a whole.
1–4	Two parameters are required, weight and growth, and the formulas vary by sex. Formulas for growth are given on pages 26 and 27 and formulas for total energy expenditure are given on page 21. The same two formulas apply for all children and adolescents aged 1–18 years. In addition, the manual recommends reducing the energy requirements of children 1–2 years of age by 7% to smooth energy requirements between children less than one year of age and children 1–2 years of age (bottom of table 4.2 on page 26 and bottom of table 4.3 on page 27).	The mean daily weights of young children aged 366–1826 days were calculated using the 2006 WHO growth standards for weight for age. Optimal growth was calculated by subtracting the mean weight of a child with the mean weight of a child of the same sex who is one day older. Using FAO formulas, daily energy requirements for children of each day of age and sex were calculated. For the 7% reduction in requirements for children aged one completed year, we applied a correction across the 12 months of the second year of life, adjusting the reduction as follows: 12 m – 14%, 13 m – 12%, 14 m – 10%, 15 m – 9%, 16 m – 8%, 17 m and 18 m – 7%, 19 m – 6%, 20 m – 5%, 21 m – 4%, 22 m – 2%, and 23 m – 0%. Daily energy requirements were averaged to obtain the average energy need for each month of life and averaged over the year as a whole.
5–9	Two parameters are required, weight and growth, and the formulas vary by sex. Formulas for growth are given on pages 26 and 27 and formulas for total energy expenditure are given on page 21. The same two formulas apply for all children and adolescents aged 1–18 years. To accommodate high and low activity levels the energy requirements are scaled up or down by 15% in line with the recommendation on page 24.	The mean monthly weights of young children aged 60 months were extracted in the same way as for the children aged 1 to 4 years. In contrast, for children aged 61–119 months only the mean monthly weight was available from the 2007 WHO growth reference for weight for age. Optimal growth was calculated by subtracting the mean weight of a child with the mean weight of a child of the same sex who is one month older. Using FAO formulas, daily energy requirements for children of each month of age and sex were calculated. We scaled these energy requirements up and down to account for activity level. Daily energy requirements were reported for each month of life and averaged over each the year of life.
10–18	Two parameters are required, weight and growth, and the formulas vary by sex. Formulas for growth are given on pages 26 and 27 and formulas for total energy expenditure are given on page 21. These same two formulas apply for all children and adolescents aged 1–18 years. To accommodate high and low activity levels the energy requirements are scaled up or down by 15% in line with the recommendation on page 24.	As there are no universal growth charts for child weight in this age range, we used the 2007 WHO growth reference for BMI for age and height for age, with adjustments for the short stature of Bangladeshis. First, the mean BMI for each month of life from 120–227 months of age was calculated from the 2007 WHO growth reference for BMI for age. Secondly, the prospective mean height for each month of life from 120–227 months of age was calculated from the 2007 WHO growth reference for height for age. As the mean height of adults using the growth reference chart is considerably taller than the height of Bangladeshi adult men and women as recorded in the 2011 BDHS (1.78 m vs. 1.63 m for men, and 1.62 m vs. 1.51 m for women), the maximal height was adjusted to the 2011 BDHS recorded level and the intermediate measures were smoothed by reducing the growth pattern to match the maximal height as given in the following formula: $x_{a,s}=\left(\left(h_{a,s}-h_{a120,s}\right)\times \frac{m_{s}}{h_{a228,s}}\right)+h_{a120,s}$ Where $x_{a,s}=$ Adjusted height of an individual of a given age and sex $h_{a,s}=$ Height of an individual of a given age and sex from the growth reference curve $h_{a120,s}=$ Height of an individual of a given sex at age 120 months from the growth reference curve $m_{s}=$ Mean height of an adult individual of the given sex as observed in the population $h_{a228,s}=$ Adult height of an individual of a given sex at age 228 months from the growth reference curve These heights were then converted to weights using the mean BMI by age in months given in the 2007 WHO growth reference, using the formula below: $w_{a,s}=b_{a,s}\times {h_{a,s}}^{2}$ Where $w_{a,s}=$ Calculated weight of an individual of a given age and sex $b_{a,s}=$ BMI of an individual of a given age and sex from the growth reference curve $h_{s}=$ height of an individual of a given age and sex from the growth reference curve Using FAO formulas, daily energy requirements for adolescents of each month of age and sex were calculated. We scaled these energy requirements up and down to account for activity level. Daily energy requirements were reported for each month of life and averaged over each the year of life.
19–29	Two parameters are required, weight and activity level, and the formula varies by sex (men's information in table 5.4 on page 41 and women's information in table 5.7 on page 44). Physical Activity level guidelines are given on page 38.	To obtain weight estimates, we used the mean BMI of adults of 19 years from the 2007 WHO growth curves (male 22.2 and female 21.4) and the adult heights obtained from the 2011 DHS (given above). These two measures were converted to adult weight using the formula given above (male 58.4 kg and female 48.9 kg), and were different than the weights recorded for this age group in the 2011 DHS (males 53.8 kg and females 48.3 kg). We used a physical activity level (PAL) of 1.55 for the sedentary group, 1.85 for the moderately active group, and 2.15 for the highly active group. Daily energy requirements were the same for all ages in this group.
30–59	Two parameters are required, weight and activity level, and the formula varies by sex (men's information in table 5.5 on page 42 and women's information in table 5.8 on page 45). Physical Activity level guidelines are given on page 38.	To obtain weight estimates, we used the same process detailed in the 19–29 years of age group including PAL levels. Calculated weights were different than the weights for this age group recorded in the 2011 DHS (males 55.0 kg and females 49.6 kg). Daily energy requirements were the same for all ages in this group.
60+	Two parameters are required, weight and activity level, and the formula varies by sex (men's information in table 5.6 on page 43 and women's information in table 5.9 on page 46). Physical Activity level guidelines are given on page 38.	To obtain weight estimates, we used the same process detailed in the 19–29 years of age group including PAL levels. Calculated weights were different than the weights for this age group recorded in the 2011 DHS (males 50.0 kg and females 43.4 kg). Daily energy requirements were the same for all ages in this group.
Other adjustments	Information on energy expenditure is taken directly from the report (no recalculation). For details on the pregnancy calculations, see section B of table 6.3 on page 59. The lactation adjustment is drawn from section 7.2.	The lactation adjustment calculates the additional energy cost of lactation to the mother as the energy requirements of the child are accounted for separately as a household member. The inefficiency of breastmilk production is given as 20% of child breastmilk intake in the FAO report. As the FAO report does not provide estimates for breastmilk intake of infants older than one year, we applied the 6–11 month values to the second year. For pregnancy, the energy requirements were taken from the energy cost of pregnancy estimated from the increment in Basel Metabolic Rate and energy deposition.

For children 0–9 years of age, the mean weight-for-age from the 2006 growth standards and 2007 growth reference curves were used with the appropriate formula, provided by FAO, to estimate the energy requirements for ideal growth. As AME are usually applied by age in months or age in years, energy need by day for children less than five years of age and energy requirements by month for children five to nine years of age were averaged into monthly and yearly values. In line with FAO recommendations, the energy requirements for school-aged children (6–9 years of age) were increased by 15% for a high level of activity, and decreased by 15% for a low level of activity [6], while no adjustment was provided by FAO for infants and children less than five years of age.

As ideal weight-for-age growth curves do not exist for adolescents and adults, and most household consumption and expenditure surveys do not record the actual heights and weights of respondents, multiple sources of information were combined to calculate the average energy requirements of adolescents and adults. We specifically chose to use actual heights of Bangladeshis but prescriptive levels for weight at these heights. This is important as height among the adult population is not assumed to be identical between populations, in contrast to the universal child growth standards. In addition, increased energy consumption during adulthood will result in adiposity.

We examined the average height among adult men and women as recorded in the 2011 Bangladesh Demographic and Health Survey (BDHS) [5]. We noted that adult heights were not substantially different for 20-year-old men and women than for 50-year-old men and women, indicating that the heights of adult men and women have not changed substantially over the period between 1980 and 2010. Because heights did not vary over this period, we used the mean height of men and women to calculate an ideal weight using the 2009 WHO growth reference curves for BMI-for-age for 19-year-olds by sex. This weight was used to estimate average energy requirements among adults using the appropriate formulas.

For adolescents, we scaled the 2007 WHO height-for-age growth reference curve to the observed average adult height in Bangladesh and calculated the ideal weight for this height using the 2007 WHO growth reference curves for BMI-for-age. In line with FAO recommendations, the energy requirements for adolescents were increased by 15% for a high level of activity, and decreased by 15% for a low level of activity [6].

The energy requirements for adults were calculated with a physical activity level (PAL) of 1.85 for a moderate level of activity, a PAL of 2.15 for a high level of activity, and a PAL of 1.55 for a low level of activity. These values are within the ranges recommended by the FAO guidelines [6].

We calculated the additional energy need of lactating women, considering that the energy requirements for infants and young children are recorded separately in household consumption surveys. To avoid double counting of both the energy expended by the lactating mother and received by the breastfeeding child, additional energy need for lactating women is limited to the energy required by the mother to convert her consumption to breastmilk. In the FAO manual this conversion loss rate for breastfeeding is estimated at 20% of energy given to the child as breastmilk. For completeness, we also included the energy costs and AME for pregnancy by trimester, though pregnancy status is often not included in HCES surveys. The calculated energy requirements for all sex and age groups were converted to AME using the energy requirement of an adult male 30 to 50 years of age, as recommended by the FAO [6].
