# Supplementary material for: Estimates of average energy requirements in Bangladesh: Adult Male Equivalent values for use in analyzing household consumption and expenditure surveys
Source: Data Brief. 2017 Jul 17;14:101–6. doi: 10.1016/j.dib.2017.07.022 (PMC5567394; doi:10.1016/j.dib.2017.07.022)
Supplement: Supplementary file 1 — Supplementary material [file mmc1.docx]

The authors declare that they have no conflict of interest.
